# Hybrid reference genome assemblies for *Leishmania (Viannia) braziliensis*, a primary agent of mucocutaneous leishmaniasis

**DOI:** 10.1128/mra.01317-24

**Published:** 2025-06-17

**Authors:** James A. Cotton, Frederik Van den Broeck, Peter J. Myler, Natalia S. Akopyants, Matthew Berriman, Stephen M. Beverley

**Affiliations:** 1 Wellcome Sanger Institute 47665, Hinxton, United Kingdom; 2 School of Biodiversity, One Health and Veterinary Medicine, University of Glasgow 3526, Glasgow, United Kingdom; 3 Department of Biomedical Sciences, Institute of Tropical Medicine 290967 , Antwerp, Belgium; 4 Department of Microbiology, Immunology, and Transplantation, Rega Institute for Medical Research, Katholieke Universiteit Leuven 54515, Leuven, Belgium; 5 Center for Global Infectious Disease Research, Seattle Children’s Research Institute, Seattle, Washington, USA; 6 Department of Pediatrics, University of Washington 271845, Seattle, Washington, USA; 7 Department of Biomedical Informatics and Medical Education, University of Washington 312757 , Seattle, Washington, USA; 8 Department of Molecular Microbiology, Washington University School of Medicine 7548, St. Louis, Missouri, USA; 9 School of Infection and Immunity, University of Glasgow 3526, Glasgow, United Kingdom; University of California Riverside, Riverside, California, USA

**Keywords:** *Leishmania braziliensis*, *Viannia*, comparative genomics, mucocutaneous leishmaniasis, Kinetoplastida, Trypanosomatidae, South America

## Abstract

We report high-quality long-read genome assemblies and annotations for two widely studied reference strains of *Leishmania (Viannia) braziliensis*, a primary agent of cutaneous and mucocutaneous leishmaniasis. These genomes should facilitate studies of animal infectivity and pathogenesis of cutaneous and severe mucocutaneous leishmaniasis.

## ANNOUNCEMENT

Species of the trypanosomatid protozoan *Leishmania* (*Viannia*) are widespread parasites of mammals within South and Central America, transmitted by biting Phlebotomine sand flies. These infections are primarily zoonotic but cause a range of human diseases, including localized or disseminated cutaneous lesions, or less frequently, disfiguring mucocutaneous disease (1, 2). *L. (Viannia*) represents an early diverging group of *Leishmania*, with numerous differences from later-diverging subgenera including development within the insect hindgut, retention of the RNA interference pathway, the frequent presence of RNA viruses, and a tendency toward mucocutaneous disease (3–9).

Here, we report two improved genome assemblies (10, 11) using long-read technology (PacBio) and hybrid assembly. *Leishmania braziliensis* M2903 and M2904 were isolated in 1975 from two patients with cutaneous lesions in Parauapebas Municipality, Brazil (12). These have been widely used in genetic studies (6, 10, 11, 13–16), with M2903 being primarily diploid and M2904 triploid, both accompanied by some degree of aneuploidy as commonly seen in all cultured *Leishmania* spp. (17). The hybrid assemblies each include 35 chromosomal contigs, as well as the maxicircle (mitochondrial/kinetoplast DNA).

Parasites cultivated in Schneider’s medium at 26°C in sealed flasks (6) were grown to the late log phase (3–4 days) and harvested by centrifugation. Nuclei were purified (18) and extracted by two rounds of gentle phenol/chloroform extraction (19), yielding genomic DNA >20 kb. This was sheared to 20–25 kb by passing through a 25 mm needle and then used to generate template libraries with v3.0 of the PacBio DNA template preparation kit with a 7 kb size selection (BluePippin, Sage Science, Beverly, MA). Libraries sequenced using the P6-C4 chemistry on a PacBio RS II platform with 3 h movies on 12 SMRT cells for M2904 (yielding 1.08 M reads, mean length 6.9 kb, and 7.6 Gb data) and 10 SMRT cells for M2903 (1.03 M reads, mean length 4.2 kb, and 4.4 Gb data). Illumina data for M2904 were generated using an amplification-free protocol (20) on an Illumina GAIIx platform (52 M paired-end reads of 76 base pairs each, total of 3.95 Gb; ENA ERS003039). For M2903, Illumina data were taken from ENA accession SRR1028154 (9.9 M paired-end reads of 100 bp each, total 994 Mb; [11]).

PacBio data were assembled using the HGAP assembler from the smrtAnalysis package (2.3.0) with an expected genome size of 20 Mb and otherwise default settings, including quality trimming. These were refined manually using PacBio reads corrected using Canu (v1.7.1) (21) and REAPR v1.0.18 (22) using default settings. This refined assembly was then polished using the untrimmed Illumina data with Pilon v1.22 (23) using default settings and with icorn2 version 0.95 from the PAGIT toolkit (24) for 15 iterations and expected insert size of 250 bp and 10 iterations and 250 bp for M2903 and M2904, respectively, using read pairs with insert sizes between 100 and 900 bp in each case. Annotation was using Companion v.1.0.2 (25) with exonerate and RATT and default kinetoplastid weights and guided by previous *L. braziliensis* M2904 gene models. Maxicircle contigs were manually circularized and annotated. Final gene numbers (see Table 1) were similar to those in other *Leishmania* genomes (10, 11).

**TABLE 1:** *L. (Viannia) braziliensis* hybrid assemblies

		*Lbr*M2903	*Lbr*M2904
World Health Organisation (WHO) code		MHOM/BR/75/M2903	MHOM/BR/75/M2904
Approximate ploidy		Diploid	Triploid
Source		Human, cutaneous	Human, cutaneous
Provenance		J. Shaw, Brazil	J. Shaw, Brazil
Publication		This work	This work
Genome assembly		GCA_964014055.1	GCA_964014045.1
Assembled annotated scaffolds		OY748431-OY748521	OY748380-OY748430
Sequence Set		CAXGYW01	CAXGYV01
Read data (add hyperlinks)		PacBio: ERS527779	PacBio: ERS527780
		Illumina: see reference 11	Illumina: ERS003039
Assembler		HGAP and manual improvement	HGAP and manual improvement
Contigs		91	51
N50 contig length		1.05 Mb	1.14 Mb
Chromosomes		35	35
Maxicircle in assembly?		Yes	Yes
Assembly size		33.49 Mb	33.51 Mb
GC content		58.01%	58.04%
Protein coding genes (including maxicircle)		8,907	9,097

## Data Availability

Annotations and assemblies are deposited in the European Nucleotide Archive (see Table 1). Single annotation files for all scaffolds in each assembly can be obtained via the ENA sequence set accessions.
